# Identification of Hürthle cell cancers: solving a clinical challenge with genomic sequencing and a trio of machine learning algorithms

**DOI:** 10.1186/s12918-019-0693-z

**Published:** 2019-04-05

**Authors:** Yangyang Hao, Quan-Yang Duh, Richard T. Kloos, Joshua Babiarz, R. Mack Harrell, S. Thomas Traweek, Su Yeon Kim, Grazyna Fedorowicz, P. Sean Walsh, Peter M. Sadow, Jing Huang, Giulia C. Kennedy

**Affiliations:** 1 Department of Research & Development, Veracyte, Inc, 6000 Shoreline Court, Suite 300, South San Francisco, CA 94080 USA; 2 Department of Surgery, Section of Endocrine Surgery, University of California San Francisco, San Francisco, CA USA; 3 Department of Medical Affairs, Veracyte, Inc, South San Francisco, USA; 4 The Memorial Center for Integrative Endocrine Surgery, Hollywood, FL USA; 5 Thyroid Cytopathology Partners, Austin, TX USA; 6 Department of Pathology, Head and Neck Pathology Division, Massachusetts General Hospital and Harvard Medical School, Boston, MA USA; 7 The Memorial Center for Integrative Endocrine Surgery, Weston, FL USA; 8 The Memorial Center for Integrative Endocrine Surgery, Boca Raton, FL USA

**Keywords:** Machine learning, Algorithm, Genomic, RNA-seq, Thyroid cancer, Hürthle, Personalized healthcare

## Abstract

**Background:**

Identification of Hürthle cell cancers by non-operative fine-needle aspiration biopsy (FNAB) of thyroid nodules is challenging. Resultingly, non-cancerous Hürthle lesions were conventionally distinguished from Hürthle cell cancers by histopathological examination of tissue following surgical resection. Reliance on histopathological evaluation requires patients to undergo surgery to obtain a diagnosis despite most being non-cancerous. It is highly desirable to avoid surgery and to provide accurate classification of benignity versus malignancy from FNAB preoperatively. In our first-generation algorithm, Gene Expression Classifier (GEC), we achieved this goal by using machine learning (ML) on gene expression features. The classifier is sensitive, but not specific due in part to the presence of non-neoplastic benign Hürthle cells in many FNAB.

**Results:**

We sought to overcome this low-specificity limitation by expanding the feature set for ML using next-generation whole transcriptome RNA sequencing and called the improved algorithm the Genomic Sequencing Classifier (GSC). The Hürthle identification leverages mitochondrial expression and we developed novel feature extraction mechanisms to measure chromosomal and genomic level loss-of-heterozygosity (LOH) for the algorithm. Additionally, we developed a multi-layered system of cascading classifiers to sequentially triage Hürthle cell-containing FNAB, including: 1. presence of Hürthle cells, 2. presence of neoplastic Hürthle cells, and 3. presence of benign Hürthle cells. The final Hürthle cell Index utilizes 1048 nuclear and mitochondrial genes; and Hürthle cell Neoplasm Index leverages LOH features as well as 2041 genes. Both indices are Support Vector Machine (SVM) based. The third classifier, the GSC Benign/Suspicious classifier, utilizes 1115 core genes and is an ensemble classifier incorporating 12 individual models.

**Conclusions:**

The accurate algorithmic depiction of this complex biological system among Hürthle subtypes results in a dramatic improvement of classification performance; specificity among Hürthle cell neoplasms increases from 11.8% with the GEC to 58.8% with the GSC, while maintaining the same sensitivity of 89%.

**Electronic supplementary material:**

The online version of this article (10.1186/s12918-019-0693-z) contains supplementary material, which is available to authorized users.

## Background

About one-third of adults have a thyroid nodule [1–3]. Physicians use thyroid ultrasonography to prioritize thyroid nodules whose size and ultrasonographic features warrant fine needle aspiration biopsy (FNAB) for the possibility of a clinically significant thyroid lesion [4–7]. An estimated 10 thyroid FNAB occur in the United States for each thyroid cancer diagnosed, suggesting that about 540,000 thyroid FNAB will occur in 2018 [8–10]. The small cytological FNAB sample is evaluated by light microscopy, and those with sufficient content are categorized by one of several nomenclature systems in order to estimate risk of malignancy [11, 12]. Nodules with benign FNAB results typically undergo clinical and thyroid ultrasound observation as the risk of cancer is < 5%, while nearly all of those with greater cancer risk historically were treated with surgery [13]. Those included the cytologically indeterminate Bethesda categories III and IV, which have an estimated risk of cancer of 11–29% [14]. Recent advances in molecular testing of cytologically indeterminate nodules have dramatically reduced diagnostic surgery among them [15, 16]. Still, cytologically indeterminate thyroid nodules with a significant Hürthle cell population occur in about 10% of all FNAB specimens [17], pose substantial cytological and molecular challenges [18], and are the focus of this research.

Hürthle cells, also known as oncocytes or oxyphil cells, are follicular-derived epithelial cells with acidophilic cytoplasm containing abundant granular (mitochondria-rich) cytoplasm [19]. Unless the cytopathologist is convinced that the Hürthle cells are part of a non-neoplastic process, such as Hashimoto thyroiditis [20], the specimen is typically categorized as cytologically indeterminate [Suspicious for Hürthle Cell Neoplasm (included within Bethesda IV), or when less Hürthle cell cellularity is present, Atypia of Undetermined Significance/Follicular Lesion of Undetermined Significance (included within Bethesda III)] [21]. While most cytologically indeterminate Hürthle cell FNAB are from benign thyroid nodules, these have historically been recommended for diagnostic surgical resection [20] because of a 9–39% risk of malignancy [18, 22, 23].

Insights into Hürthle cell carcinomas are growing, with recent investigations reporting alterations in nuclear and mitochondrial genomes and enriched genomic instability, which differ from the genomic profiles of non-Hürthle neoplasms [24, 25]. Still, mitochondrial and classic DNA mutations, and other cytological, radiological, and laboratory approaches have shown low sensitivity in detecting carcinomas among Hürthle cell FNAB, and imperfect specificity in differentiating benign from malignant nodules [20, 26, 27]. In 2011, the Afirma® gene expression classifier (GEC) was developed as a cancer “rule-out” test for cytologically indeterminate nodules with the intention that GEC benign samples with a low risk of malignancy could undergo clinical observation similar to cytologically benign nodules [15, 16, 28]. While many FNAB contain Hürthle cells from non-neoplastic nodules, it was also recognized that differentiating Hürthle adenomas (HCA) from Hürthle carcinomas (HCC) was a major challenge [29]. With an overall goal of an accurate benign GEC test result, a Hürthle cassette was inserted upstream of the main GEC classifier. The Hürthle cassette erred on the side of caution by identifying all samples with strong Hürthle cell neoplastic mRNA signatures as suspicious, while allowing the remainder to pass through to the main GEC classifier for final evaluation as molecularly benign or suspicious. The impact of this process is seen in the 2012 clinical validation study in which only 19% of HCA received an overall benign result, compared to 58% of non-Hürthle adenomas [15]. In practice, centers whose Hürthle FNAB included more non-neoplastic samples, or samples that passed through the Hürthle cassette, received an overall benign result among Hürthle FNAB in about one-third of their samples [30, 31], whereas others reported benign results less often [32, 33]. While the accuracy of the benign result among Hürthle FNAB remained high, the low benign call rate diminished the cost-effectiveness of the test amongst this sample type [33].

Recently, the GEC test was migrated from a microarray mRNA expression platform to a next-generation RNA sequencing platform, which provided access to RNA transcriptome expression and sequencing of nuclear and mitochondrial RNA; and detection of genomic copy number, including loss-of-heterozygosity (LOH). We coupled this increased genomic content with enhanced bioinformatics and machine-learning (ML) strategies to maintain high test sensitivity with improved overall test specificity. A key objective was to improve the specificity in classification of FNAB containing Hürthle cells. This enhanced test is the Afirma Genomic Sequencing Classifier (GSC) [34]. Here, we describe the development and validation of a Hürthle cell Index (HI) to detect FNA samples with Hürthle cell features in all FNA samples tested with an additional Hürthle cell Neoplasm index (NI) to further score only HI positive cases and separate them into neoplastic and non-neoplastic categories. These two indices enable the test to function without input from physician cytological interpretation and automatically interfaces with the core GSC classifier to render an overall GSC benign or suspicious result on every FNAB specimen. The result is a dramatic improvement of specificity among Hürthle cell neoplasms from 11.8% with the GEC to 58.8% with the GSC, while maintaining the same sensitivity of 89% [34]. This improvement in specificity substantially increases number of highly accurate benign result among Hürthle cell FNAB, safely saving patients with these challenging nodules from diagnostic thyroid surgery.

## Methods

### Study design

Three different types of thyroid samples were utilized for this study. The first group (Cyto-Hürthle) included FNAB for developing Hürthle cell and Hürthle cell Neoplasm indices, where detailed cytologic features were curated by expert thyroid cytopathologists using microscopic examination from FNAB. Hürthle cell-specific cytology class labels were assigned based on the presence or absence of Hürthle cells and potentially neoplastic features. The second group was comprised of FNAB for developing the Afirma Benign/Suspicious (B/S) classifier, where histopathology diagnoses were available. Two subsets were separately analyzed, as described in [34]: (1) a subset of samples used for training the Afirma GSC and (2) 191 samples used for the validation of Afirma GSC. The third group used fresh, frozen thyroid surgical tissues with histopathology diagnoses, including 12 Hürthle cell and 43 non-Hürthle cell cases, for examining copy number variation (CNV) and LOH using the Affymetrix CytoScan platform.

### Identifying cytopathology samples for review

The Veracyte database was queried to identify 285 FNAB where Hürthle cell features were noted in the initial cytological reading of the case. An additional 272 FNAB where no Hürthle cell features were noted were also selected. These cases were subject to blinded re-review by a panel of 3 cytopathologists. Features examined were: cellularity, proportion of Hürthle cells, Hürthle cell morphology, Hürthle cell maturation spectrum, and presence or absence of colloid. Based on these features, four classes of samples were generated (See Additional file 1: Figure S1 for representative images): 1. Hürthle cell positive, Neoplasm positive (H + N+); 2. Hürthle cell positive, Neoplasm negative (H + N-); 3. Hürthle cell negative, Neoplasm positive (H-N+); 4. Hürthle cell negative, Neoplasm negative (H-N-). This analysis yielded 318 samples, including 119 Hürthle cell-negative and 199 Hürthle cell-positive samples. Of the 199 Hürthle cell-positive samples, 27 were identified as Bethesda II and were therefore labelled Neoplasm-negative, while 71 were Bethesda IV and were therefore labelled Neoplasm-positive. Samples were de-identified prior to cytopathology re-review prior to RNA Access library preparation.

### Affymetrix CytoScan

Thyroid tissue DNA was extracted with the AllPrep Micro kit (Qiagen, Hilden, Germany) and quantitated with the Pico Green dsDNA kit (Thermo Fisher) on a Tecan Infinite Pro 200 plate reader (Tecan, Männedorf, Switzerland). DNA (125 ng) was used as input into the CytoScan HD array kit (Affymetrix, Santa Clara, CA) and the samples were processed according to the manufacturer’s protocol. Cel files were input into the Affymetrix Chromosome Analysis Software (ChAS) and Copy Number and SNP outputs were analyzed for LOH and other CNVs.

### RNA library preparation and next-generation sequencing

Samples were processed as described [34]. Briefly, 15 ng of total RNA was input into a Microlab STAR (Hamilton, Reno, NV) automated version of the TruSeq RNA Access Library Preparation Kit (Illumina, San Diego, CA). Libraries were sequenced on the NextSeq 500 (Illumina, San Diego, CA) using paired-end 2 × 76 cycle reads.

### RNA sequencing pipeline, feature extraction, and quality control

RNA-seq data were processed as described [34]. Raw sequencing data was aligned to human reference genome assembly 37 using STAR aligner. Normalized expression levels were obtained using variance stabilizing transformation (VST) from the DESeq2 package [32]. The gene-wise dispersion parameter was estimated by the ‘local fit’ method. Genome-wide variants were identified using the GATK variant calling pipeline. Samples that did not satisfy the minimum in-house sequencing QC metrics were excluded from downstream analyses.

### Feature engineering

#### Loss-of-heterozygosity (LOH) statistic

We developed a LOH statistic at the chromosome and genome level using genome-wide variants. The statistic quantifies the magnitude of LOH by calculating the proportion of variants that have a variant allele frequency (VAF; fraction of reads carrying the alternative allele) away from 0.5 (< 0.2 or > 0.8) after pre-filtering of variants with a VAF exactly at one. For the genome-level LOH statistic calculation, the mitochondrial genome and X and Y chromosomes were excluded. The details of the LOH statistic calculation are shown in the formulas below, where “*n_loss_het*” is the number of variants with a VAF far away from 0.5 (< 0.2 or > 0.8), and “*n_all_het*” is the total number of potentially heterozygous variants. LOH statistic was calculated both for each chromosome (referred as chromosome-level LOH) and for the entire genome (referred as the genome-level LOH).

$$ LOH=\frac{n\_ loss\_ het}{n\_ all\_ het} $$

$$ n\_ loss\_ het={\sum}_{i=1}^N{\displaystyle \begin{array}{c}1\  if\ 0< vaf<0.2\  or\ 0.8< vaf<1\\ {}0\end{array}} $$

$$ n\_ all\_ het={\sum}_{i=1}^N{\displaystyle \begin{array}{c}1\  if\ 0< vaf<1\\ {}0\end{array}} $$

Fifty-four tissue samples have LOH measured by both Affymetrix CytoScan and RNA-seq. Concordance between these two methods is shown in Additional file 1: Figure S2. These data correlated well on the samples with high level LOH (> 0.2 by Affymetrix CytoScan), all of which were Hürthle samples.

#### Mitochondrial features

Mitochondrial genes were captured during RNA Access library preparation and the same experimental procedures and bioinformatic sequencing pipelines were applied as described in previous sections. In total, there are 13 protein coding genes, and transcripts from all 13 were captured by the sequencing assay. Exploratory data analysis revealed all 13 genes showed differential expression levels between Hürthle negative and positive groups. Therefore, all mitochondrial genes were included in the gene feature set to undergo feature selection in downstream classifier development.

### Hürthle cell index (HI) development

A total of 318 FNA samples (199 Hürthle cell + and 119 Hürthle cell -) were used to develop a Hürthle cell Index (HI), which is a binary classifier, determining if a sample is Hürthle cell + or Hürthle cell -. Ten-fold cross validation was performed to estimate the training performance, and the final model was built on all samples. Classifier development comprised three sequential steps: (1) differential expression analysis on 21,162 genes, using a statistical software package, edgeR [35], (2) selection of top-ranked genes with a FDR-adjusted *p*-value < 0.05 and expression fold-change (log2 scale) > 1.5, (3) optimizing parameter setting of multiple state-of-the-art machine learning algorithms with nested cross-validation. The algorithms we tested include support vector machine (SVM), elastic net, random forest, as well as SVM with asymmetrical cost to account for class imbalance. Hyperparameter tuning was performed in the inner layer; while the performance evaluation was performed in the outer layer holding out 10% of samples for each fold. SVM was selected due to its optimal cross-validated performance. The cost-parameter tuning for SVM was performed on a grid of (1e-04, 0.001, 0.01, 0.05, 0.1, 1, 5, 10). The best parameter selected for the final model was 0.001, and the associated number of support vectors was 106. Based on these parameters, the final SVM model was established using the ‘svmLinear’ method from the ‘caret’ R package [36] with all training samples and 1408 genes selected from the differential expression analysis.

### Hürthle cell neoplasm index (NI) development

Among the 199 Hürthle cell samples used for HI development, 98 were further grouped into Neoplasm+ (*n* = 71) and Neoplasm- (*n* = 27) and used for Neoplasm Index (NI) training. NI is a binary classifier, determining if a Hürthle cell + sample is Neoplasm+ or Neoplasm-. Algorithm training for the NI was carried out similarly to the training for the HI but included novel LOH statistics as features. For the final model, 2041 genes were selected from the differential expression analysis. In addition, 15 chromosome-level LOH statistics (chromosomes 1, 2, 3, 4, 5, 6, 8, 9, 11, 13, 14, 15, 16, 18, and 19) and genome-level LOH were included as features for model training. The SVM was then built similarly to HI training on the same cost-parameter grid. The best parameter selected for the final model was 0.001, and the associated number of support vectors was 51.

### Integrating Hürthle and Hürthle neoplasm indices into the Afirma GSC B/S classification workflow

**Fig. 1:**
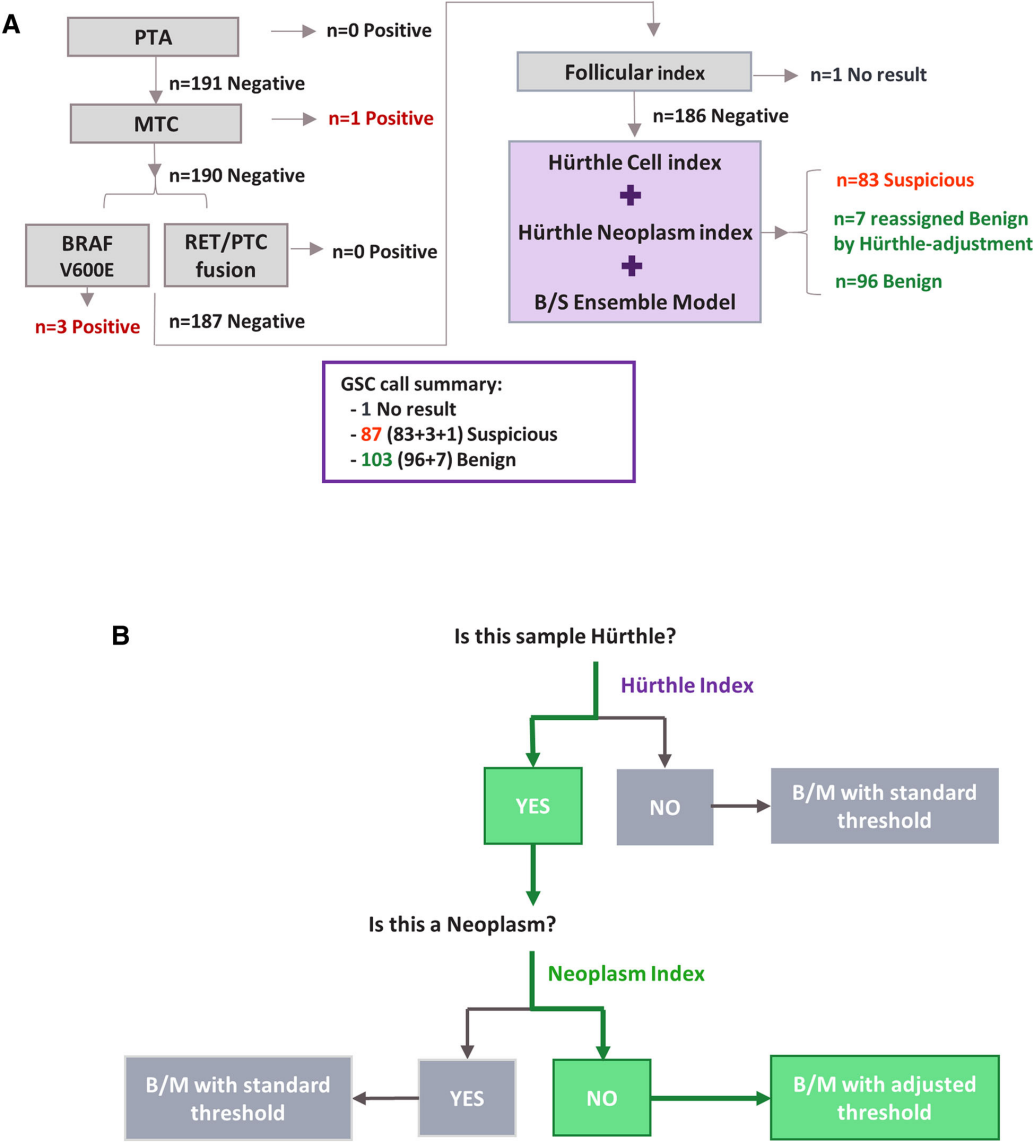
The Afirma GSC Algorithm Workflow. **a** A diagram of the Afirma GSC workflow with the validation cohort outcomes listed. **b** Nested strategy for Hürthle classification. Samples are first examined by the HI classifier. HI+ samples are passed to the NI classifier. NI- samples are subject to an adjusted threshold for the main B/S classifier

The Afirma GSC classification workflow (Fig. 1a) begins with four upstream classifiers handling special thyroid FNA entities (Parathyroid Adenoma (PTA), Medullary Thyroid Carcinoma (MTC), BRAF V600E, and RET/PTC1 and RET/PTC3 fusions). It then uses the ensemble B/S model to classify a majority of samples as GSC benign or suspicious, as described previously [34]. The HI and NI are integrated with the ensemble model to increase overall classification performance (Fig. 1b).

There are three mechanisms for arriving at the GSC Benign versus Suspicious binary outcome for a given sample:

1. The result is GSC Benign if the ensemble B/S score is lower than the nominal threshold; otherwise
2. The result is initially GSC Suspicious but can be reassigned to a benign call by “Hürthle-adjustment” if the sample is predicted as Hürthle cell Index-positive (HI+), and Neoplasm Index-negative (NI-), and the ensemble B/S score is lower than the Hürthle-adjusted threshold; otherwise
3. The result is GSC Suspicious.

The three types of outcomes are described with mathematical formulae as follows. For a given sample *i*, we denote the scores from HI and NI, and the ensemble B/S classifier as *H*_*i*_, *N*_*i*_, and *BS*_*i*_, respectively. We denote the thresholds for HI and NI as *t*_*H*_ and *t*_*N*_, respectively. For the ensemble B/S score, two thresholds exist; one is the nominal threshold, *t*_*BS_nominal*_, and the other is an increased threshold to handle Hürthle cell-positive, Neoplasm-negative cases. The latter is referred to as a “Hürthle-adjusted” threshold and denoted as *t*_*BS_Hürthle*_. The binary call outcome of the sample will be:

1. GSC Benign, if *BS*_*i*_ < *t*_*BS_nominal*_; otherwise
2. Initially GSC Suspicious, but reassign to GSC Benign if *H*_*i*_ > *t*_*H*_, and *N*_*i*_ < *t*_*N*_,and *t*_*BS_nominal*_ ≤ *BS*_*i*_ < *t*_*BS_Hürthle*_; otherwise
3. GSC Suspicious

The integration of HI and NI into Afirma GSC workflow aims to “rescue” truly benign Hürthle cell-containing samples by actively reassigning these samples to GSC benign that would otherwise have been called GSC Suspicious.

### Determining thresholds during algorithm development to maximize overall performance

Multiple factors were considered in determining thresholds for individual HI and NI, as well as the Hürthle-adjusted B/S threshold. First, cross-validation training performance of HI and NI were evaluated against the cytology label. Second, we examined the concordance of predicted Hürthle cell and Neoplasm status with the histopathology diagnosis using samples from the Afirma GSC B/S training set. Finally, the potential gain in the overall Afirma GSC performance due to Hürthle-adjustment was assessed. Due to the complexity of including two types of datasets (one focused on cytologic features, and the other on histopathology labels) and integration of three separate classifiers, complex dynamic parameter optimization was engaged with extensive multi-dimensional grid search to examine the tradeoff in the sensitivity and specificity for each classifier alone, and in combination. The final thresholds were chosen by optimizing overall Afirma GSC B/S specificity, while maintaining a high sensitivity (> 90%), by enabling high performance in both HI and NI.

## Results

### Identifying mitochondrial features in Hürthle positive samples

**Fig. 2:**
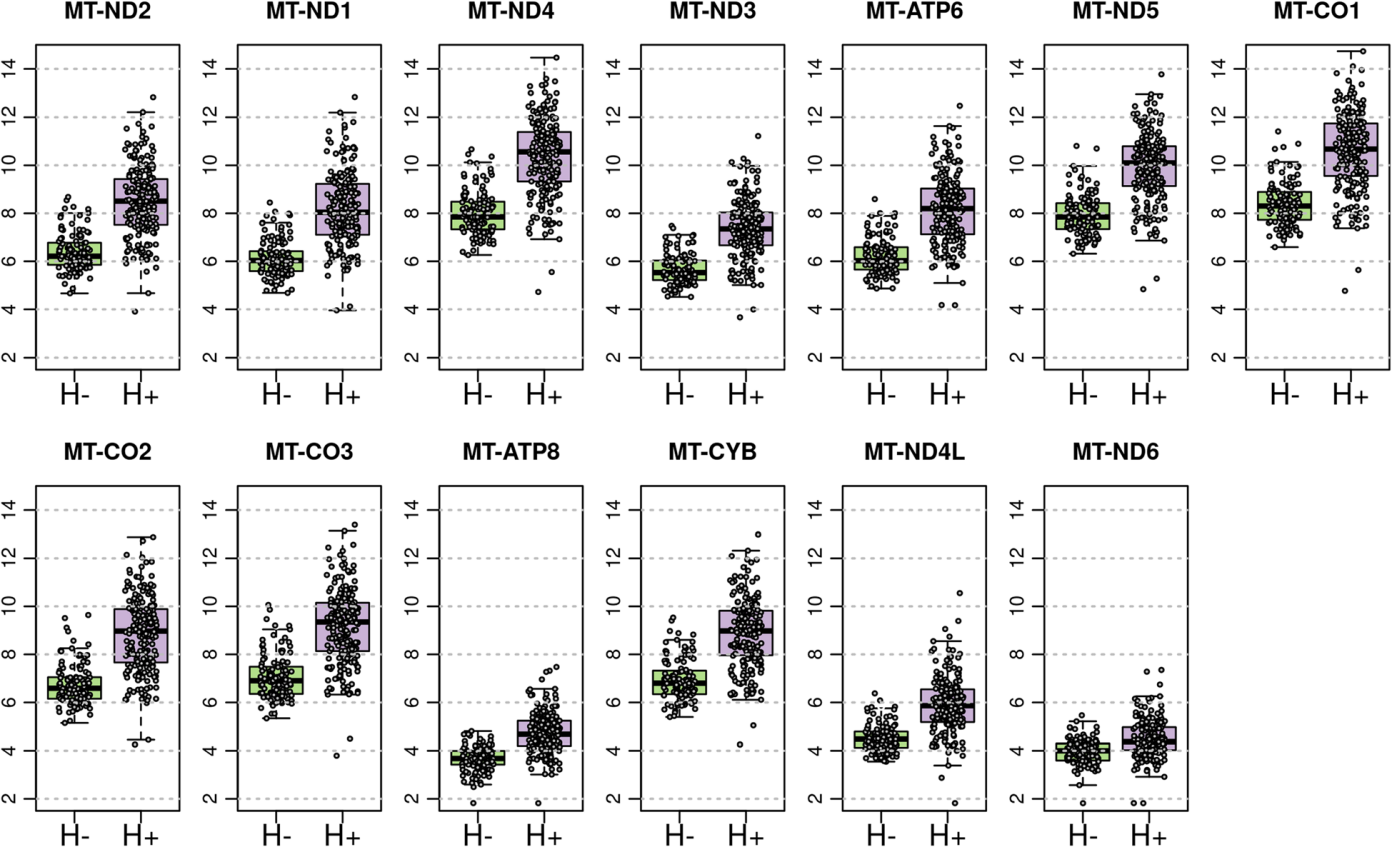
Mitochondrial expression in cytopathology Hürthle positive (H+) and negative (H-) cohorts. Shown are the 13 mitochondrial transcripts present in RNA-seq data. Each transcript shows a boxplot of expression values for Hürthle negative (H-) and Hürthle positive (H+), respectively

One prominent microscopic feature observed in Hürthle cell-positive samples, visualized by both cytopathology and histopathology, is the inherent increased intracytoplasmic mitochondria in Hürthle cells [19]. We sought to determine if there was a genomic signature associated with the high mitochondrial content noted in Hürthle cells. Analyzing differential expression between Hürthle cell positive and Hürthle cell negative samples revealed that all 13 mitochondrial transcripts were observed with an FDR-adjusted *p*-value of < 0.05 (Fig. 2). Therefore, the elevated number of mitochondria observed microscopically can be detected genomically using RNA Sequencing.

### Identifying LOH features in Hürthle positive, neoplasm positive samples

**Fig. 3:**
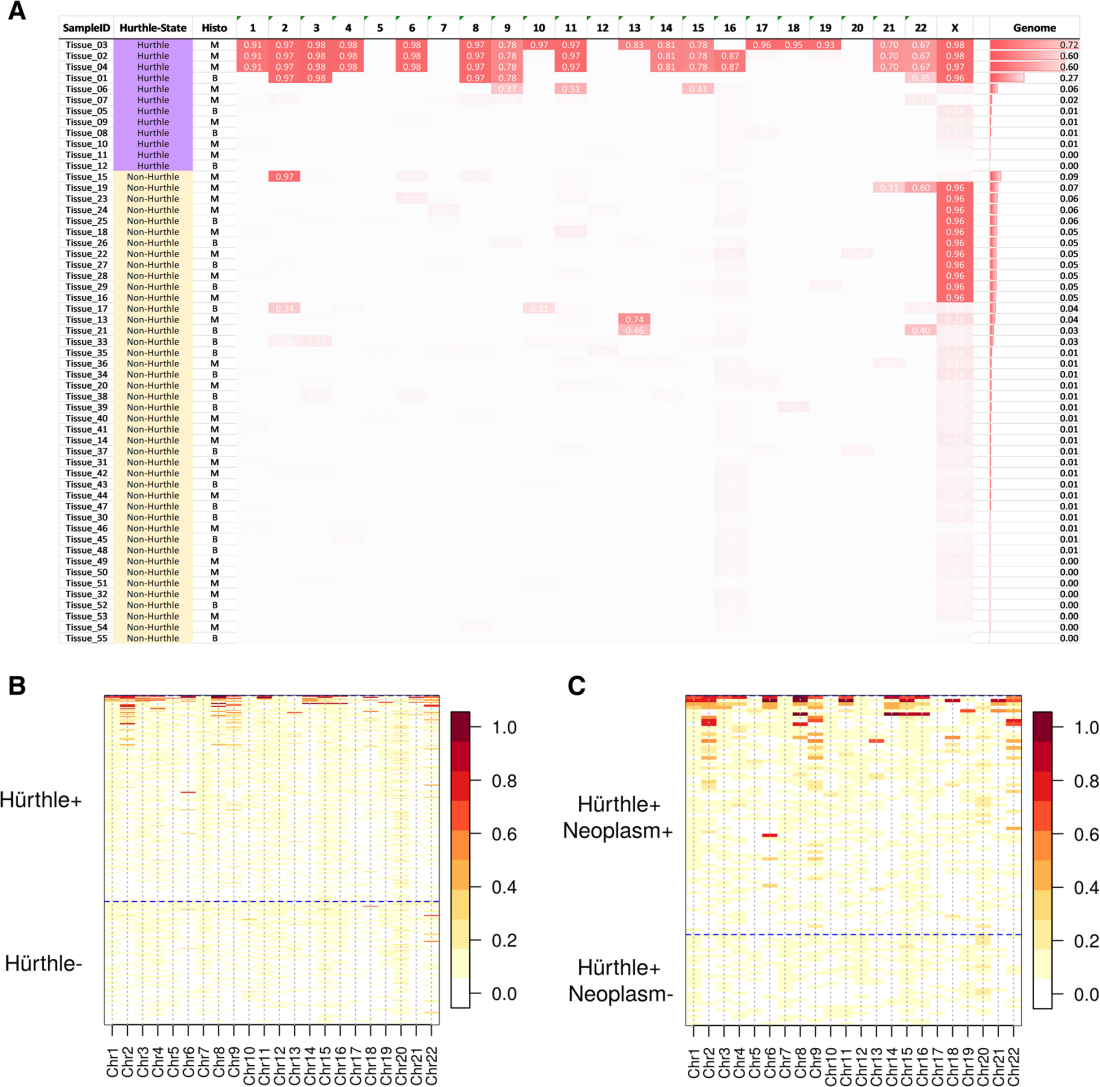
Loss of Heterozygosity in Hürthle positive samples. **a** Affymetrix CytoScan array data on Hürthle tissues vs. non-Hürthle normal tissues. Each column represents one chromosome and each row represents one sample. The value in each cell is the proportion of the chromosome displaying LOH for a given sample. The samples are sorted in descending order for genome-wide LOH. **b** Chromosome-level LOH data from RNA-Seq Hürthle positive and Hürthle negative samples. Above the horizontal dashed line are Hürthle positive samples, below the dashed line are Hürthle negative samples. **c** Chromosome-level LOH data from RNA-Seq Hürthle positive, Neoplasm positive or Neoplasm negative samples. Above the horizontal dashed line are Hürthle positive, Neoplasm positive samples, below the dashed line are Hürthle positive, but Neoplasm negative samples. For both **(b)** and **(c)** the LOH scale is to the right, with red indicating more LOH

We next sought to identify genomic features associated with Hürthle cell positive, neoplasm positive samples. Previous reports have identified copy number changes in Hürthle subtypes [26, 37, 38]. We performed genome-wide DNA copy number analysis on thyroid tissues and observed extensive LOH in many Hürthle tissues, while relatively little LOH was observed in non-Hürthle tissues (See Fig. 3a). Interestingly, the LOH was primarily, but not exclusively, enriched in Hürthle cell carcinomas (See Additional file 1: Figure S3 for example of LOH in various tissues).

Based on these DNA findings, we sought to recapitulate the LOH signal in RNA-seq data, utilizing SNPs called from expressed genes. Because RNA-seq data is limited to the exome, only chromosome-wide and genome-wide LOH were examined. We compared the CytoScan DNA data to RNA-seq data for the tissues described above and found that the genome-wide LOH signal is similar between two platforms (Additional file 1: Figure S2). Examination of LOH data from RNA-seq at the chromosome level shows that LOH is predominantly observed in Hürthle cell positive samples (Fig. 3b), although not all Hürthle cell positive samples show elevated LOH. Next, the LOH signal was examined for neoplasm positive versus neoplasm negative in the context of Hürthle cell positive samples. Only Hürthle positive, neoplasm positive samples show extensive LOH across multiple chromosomes (Fig. 3c). These data show that the LOH signal can be detected in RNA-seq data, and that it is strongly correlated with Hürthle cell positive, neoplasm positive samples.

### An expression-based classifier with mitochondrial features to separate Hürthle from non-Hürthle

**Fig. 4:**
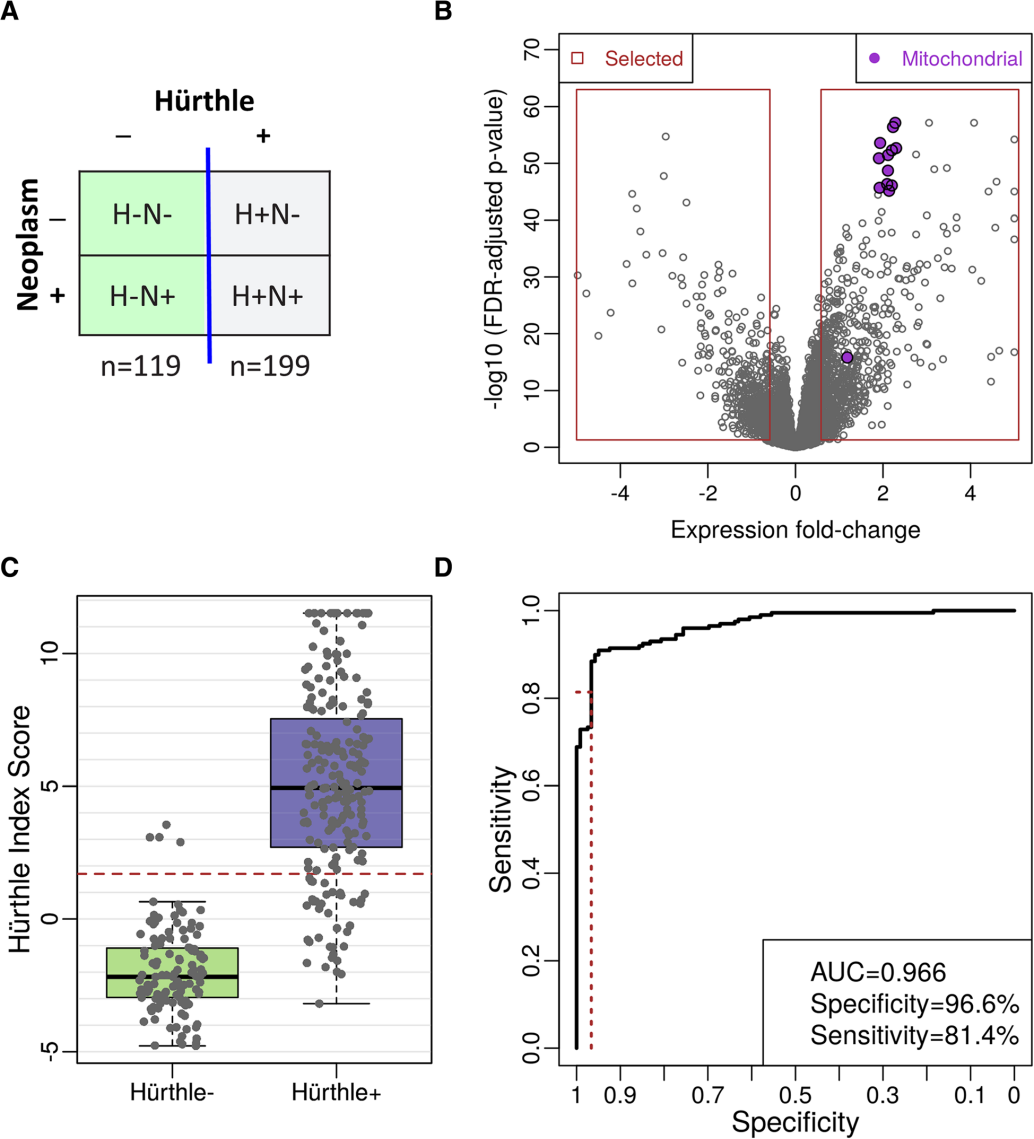
Hürthle Classifier Cross Validation Performance. **a** Samples used in the Classifier development. Hürthle and Neoplasm labels are defined by cytopathology. **b** Volcano plots of differential expression. Fold-change (log2 scale) is plotted on the x-axis, and FDR-adjusted *p*-values are plotted on the y-axis. Mitochondrial genes are shown in purple. **c** Hürthle Index Score. Red dashed line indicates the cut-off for HI+ vs. HI-. The green boxplot represents the score for cytopathology Hürthle negative samples and the purple boxplot represents cytopathology Hürthle positive samples. **d** ROC curve showing classifier performance. The red-dashed lines indicate performance at the selected cutoff

We used expression levels, including mitochondrial features, to develop a classifier that separates Hürthle cell-positive FNAB from Hürthle cell-negative FNAB (Fig. 4a and b). Classifier score distribution was wider for the Hürthle cell positive case, with a much tighter distribution for Hürthle cell negative cases (Fig. 4c). Cross-validation performance showed a high AUC (0.966) with very high specificity (96.6%) and high (81.4%) sensitivity (Fig. 4d).

### An expression-based classifier with expressed LOH features to separate Hürthle/neoplasm positive from negative

**Fig. 5:**
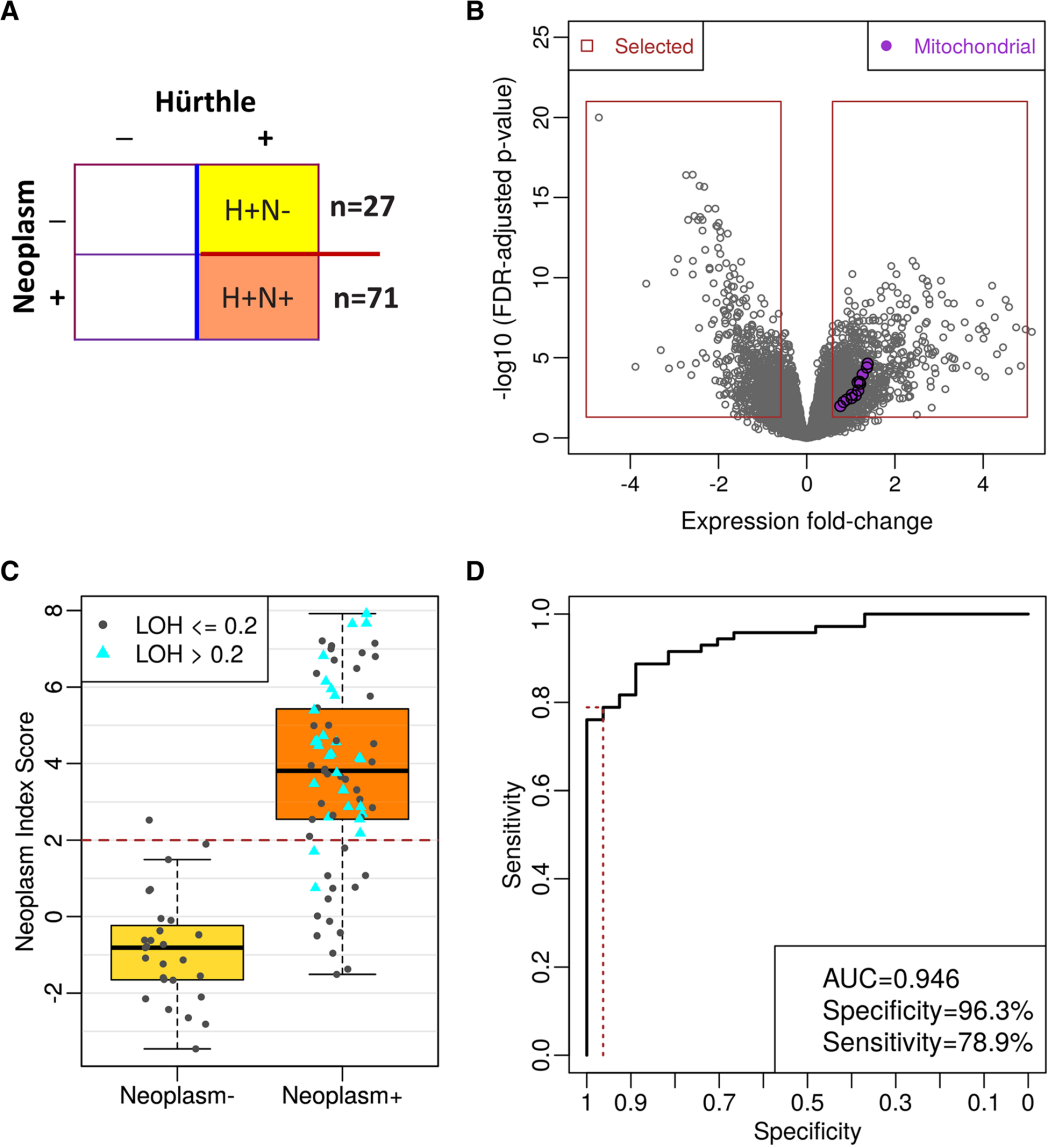
Neoplasm Classifier Cross Validation Performance. **a** Samples used in the Classifier development. Hürthle and Neoplasm labels are defined by cytopathology. Note that all samples are Hürthle positive. **b** Volcano plots of differential expression. Fold-change (log2 scale) is plotted on the x-axis, and FDR-adjusted p-values are plotted on the y-axis. Mitochondrial genes are shown in purple. **c** Neoplasm Index Score. Red dashed line indicates the cut-off for NI+ vs. NI-. Cyan triangles indicate genome-wide LOH positive. **b** ROC curve showing classifier performance. The red-dashed lines indicate performance at the selected cutoff

We developed a classifier from 27 neoplasm-negative and 71 neoplasm-positive samples, all Hürthle cell-positive (Fig. 5a and b). The neoplasm classifier score distribution is wider for the neoplasm positive samples than the neoplasm negative samples (Fig. 5c), with all but two LOH-positive samples classified as neoplasm positive (blue triangles, Fig. 5c). Cross-validation performance revealed a high AUC (0.946), with 96.3% specificity and 78.9% Sensitivity (Fig. 5d).

### Integrating the Hürthle and neoplasm classifiers into the Afirma genomic sequencing classifier algorithm workflow

The Afirma GSC includes multiple classifiers to identify key factors related to thyroid nodule malignancies [34]. Figure 1a shows the overall Afirma GSC algorithm workflow Fig. 1b shows a description of the Hürthle cell and Neoplasm algorithm workflow.

**Fig. 6:**
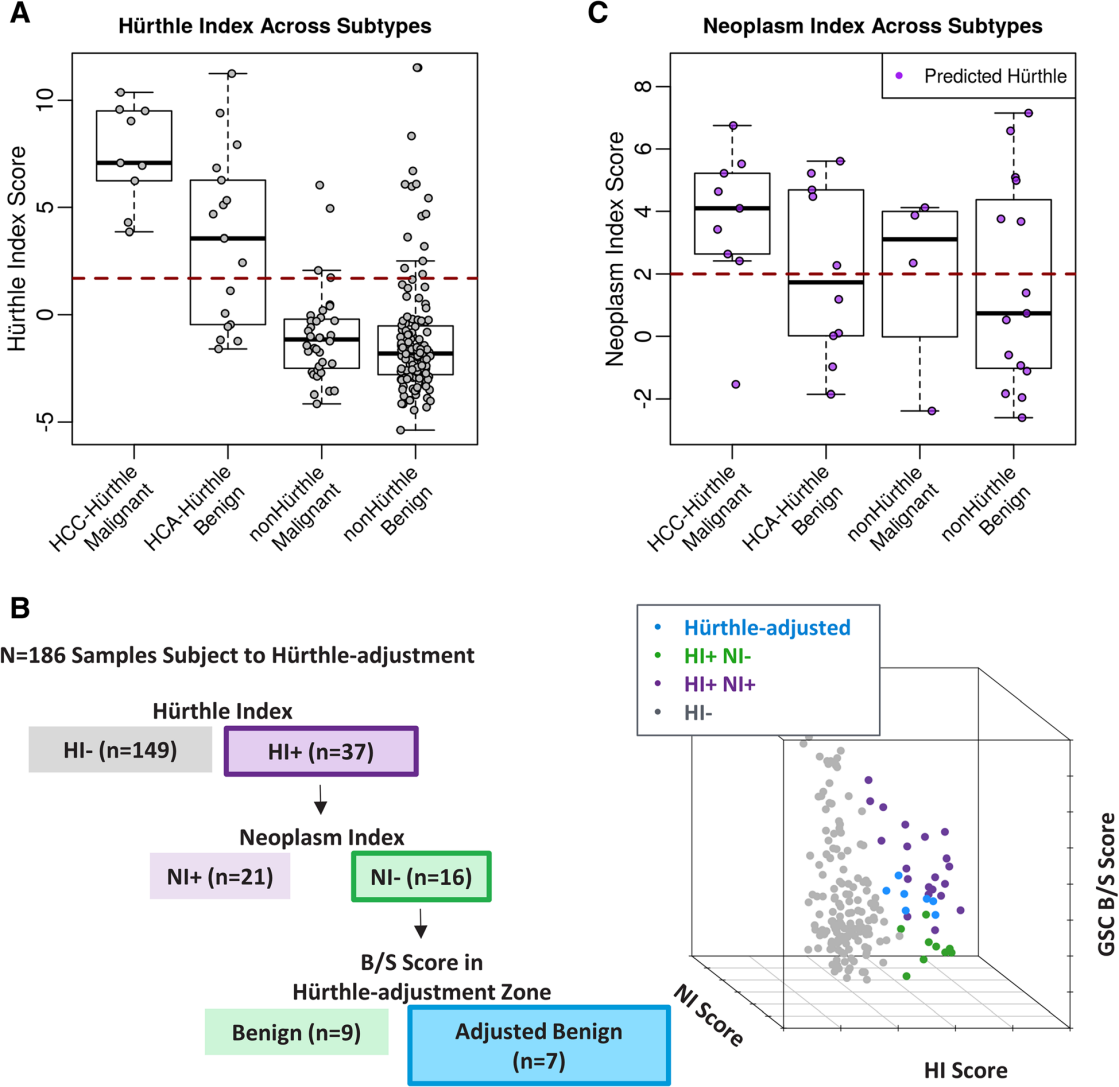
Hürthle and Neoplasm Scores from the Afirma GSC Validation Cohort. **a** HI classifier scores for the validation cohort. Red dashed line indicates cutoff for HI+ vs. HI-. HI score distribution is plotted as boxplot with individual sample values for the four groups separately: Hürthle Cell Carcinoma “HCC”, Hürthle Cell Adenoma “HCA”, non-Hürthle histopathology malignant “nonHürthle Malignant”, non-Hürthle histopathology benign “nonHürthle Benign”. **b** The combination of the main B/S, Hürthle, and Neoplasm Indices. Gray points are HI- samples. Purple points are HI+, NI+. Green points are HI+, NI-. Blue dots are HI+, NI- samples that were subject to the adjusted cutoff. **c** NI classifier scores for HI+ samples from the validation cohort

We examined the HI and NI scores for the validation cohort, using histopathology as truth, revealing that most samples are correctly classified (Fig. 6a and b). All 9 HCC samples and 10/17 HCA samples are classified as Hürthle cell positive (Fig. 6b). One HCC sample was erroneously classified neoplasm negative (Fig. 6c). It is noteworthy that this sample was a false negative in both the GEC [15] and GSC [34] validation cohorts, and this sample was characterized by several rounds of discordant histopathological diagnoses. Five of ten HI positive HCA samples were classified neoplasm negative (Fig. 6c). Seven samples were rescued by the Hürthle-adjusted threshold (Fig. 6b). Six samples were benign, including 2 HCA, 2 BFN, 1 CLT, and 1 FA. One sample was malignant, thereby resulting in one false negative. Because the HCC false negative was called benign by the B/S classifier at the nominal threshold it was not subjected to the Hürthle-adjusted threshold. Combining the B/S classifier with the HI and NI resulted in a significant performance gain in Hürthle subtypes, with specificity increasing from 11.8% with the GEC to 58.8% with the GSC.

## Discussion

Hürthle cells are follicular-derived epithelial cells with acidophilic cytoplasm. These oncocytes, or oxyphil cells, occur in multiple tissue types and are characterized by abundant, mitochondria-rich cytoplasm [19]. Known as Hürthle cells when found in the thyroid, this cellular transformation exists along a continuum, and in the thyroid is thought to result from high oxidative stress and reactive oxygen species [19]. How and if this process relates to tumorigenesis is unknown. Almost all benign and malignant thyroid neoplasms have a Hürthle cell counterpart, with the exception of anaplastic thyroid cancer [19]. Hürthle cells are associated with hyperplasia (multinodular adenomatous goiter), chronic inflammation (Hashimoto’s thyroiditis), benign neoplasia (HCA) and malignant neoplasia (HCC) [19]. Hürthle cells have long challenged cytologists to accurately allocate such FNAB specimens into definitively benign or malignant categories [20]. To not miss cancer, most nodules not categorized as benign have historically undergone surgical resection. Thus, Hürthle cell cytology has contributed to the costs, morbidity, and occasional mortality of diagnostic thyroid surgery [16].

Here we present the detailed development of two classifiers to address the possibility that a cytologically indeterminate FNAB specimen would contain Hürthle cells. The two classifiers interface with a third classifier (the core GSC classifier), whose overall specificity was improved compared to its predecessor (the GEC), and together this trio of classifiers automatically assess the molecular signature of every FNAB specimen presented to them.

Key to understanding how the two Hürthle classifiers function is to recognize that Hürthle cells are found among both neoplastic and non-neoplastic processes, and that in the absence of neoplasia, the specimen should be benign. The HI classifier first determines if the specimen contains Hürthle cells. It does this using 1408 differentially expressed genes, including 13 mitochondrial transcripts. If this HI classifier is negative, then the core GSC classifier renders a benign or suspicious result (Fig. 1). If the HI classifier is positive for Hürthle cells, then the NI classifier determines if the specimen is neoplastic, using 2041 differentially expressed genes and a novel LOH statistic. If neoplastic, then the core GSC classifier renders a benign or suspicious result. However, if the NI suggests the absence of neoplasia, then the core GSC classifier uses an adjusted threshold that allows more of these samples to receive a GSC benign result. This adjusted threshold is justified based on the absence of neoplasia. While the NI sensitivity is high, it is not perfect, and a neoplastic specimen could be falsely deemed non-neoplastic. For this reason, the specimen is not automatically given a final benign result, but rather must still pass the core GSC classifier with an adjusted (more tolerant) benign versus suspicious threshold, rather than having the threshold removed completely. Among the FNAB specimens derived from a prospective, multicenter, and blinded cohort of cytologically indeterminate thyroid nodules used to validate the GEC, and subsequently the GSC, an additional 4% of samples were given a final benign result due solely to the two Hürthle classifiers. These “rescued” samples represent 19% of those deemed “Hürthle” by the HI classifier, and 44% of those deemed “not neoplastic” by the NI (Fig. 6b). These results are similar to what we have seen subsequently in the Veracyte CLIA laboratory. In its first 6203 cytologically indeterminate specimens, 5779 specimens passed all quality control requirements and received a final result, including 3% BRAFV600E classifier positive, 0.6% parathyroid classifier positive, 0.4% RET/PTC1 or RET/PTC3 fusion positive, and 0.3% medullary thyroid cancer classifier positive. Of the remaining 5552 specimens, 21% were positive by the HI classifier, 56% of them were deemed negative by the NI classifier which invoked the adjusted core GSC classifier threshold through which 14% of the HI positive but NI negative specimens received a final benign result due to the adjusted threshold (unpublished results). The impact of the coordinated trio of classifiers described here is now being reported among independent real-world clinical experiences. In one report, the authors state that 21% of their Bethesda III and IV cytologically indeterminate FNAB were Hürthle cell-dominant. Among them, only 18% of 107 cytologically Hürthle cell-containing FNAB received a benign result by GEC, compared to 67% of 18 Hürthle cell-containing FNAB with GSC (*p* < 0.0001) [39]. Similar, independent experiences support this substantial increase in the GSC benign call rate and accuracy of this classifier trio [40, 41].

The strength of this work includes the incorporation of expert cytopathology to establish “truth” labels for training the two Hürthle classifiers. The decision cut-off for the HI classifier results in very few non-Hürthle cell samples being falsely identified by the Hürthle cell index as “Hürthle”, but a larger fraction of truly Hürthle cell samples being missed. These HI false negative samples lose the opportunity to be potentially rescued by the adjusted core GSC classifier threshold. We consider this a safer mode of failure. The high specificity of the NI classifier leads to high accuracy in samples called neoplasm positive; the lower sensitivity translates to approximately 30% of truly neoplastic (but not necessarily malignant) samples being falsely identified by as non-neoplastic and therefore invoke the more tolerant GSC classifier threshold. Despite this risk, we demonstrate in clinical validation a preserved high sensitivity among our Hürthle cell neoplasms when the entire system performance is considered. Perhaps the greatest limitation to differentiating benign from malignant Hürthle cell nodules is the imperfection of gold-standard surgical histology benign or malignant “truth” labels. Low concordance of truth labels among these specimens by expert surgical pathologists is well-known [29, 42], and even perfectly trained classifiers can only carry forward this imperfection, but they cannot correct it. Until improved “truth” labels are accepted, this barrier to improved classification will remain. It is noteworthy that we observed significant LOH among a fraction of both histologically benign and malignant Hürthle cell neoplasms. Whether or not the genomic instability of LOH should represent a pre-malignant, or carcinoma in-situ, is unknown and their natural history is unknown since these neoplasms were all surgically resected in our training and validation cohorts.

## Conclusions

Three coordinate classifiers were developed to address cytologically indeterminate Hürthle cell thyroid FNAB using ML algorithms which harness the enriched genomic content from RNA-sequencing, including mRNA expression from differentially expressed nuclear and mitochondrial genes, and a novel LOH statistic. As an adjunct to clinical judgment, this trio of classifiers empowers physicians to reduce unnecessary diagnostic thyroid surgery among these most challenging cytological specimens: an action that directly improves patient safety, saves healthcare costs, and enhances quality of life.

## Additional file

Additional file 1: **Figure S1.** Representative cytopathology images of Hürthle and Neoplasm samples. (a) Hürthle negative, Neoplasm negative sample. Bethesda II FNA. (b) Hürthle positive, Neoplasm negative sample. Bethesda II FNA. (c) Hürthle negative, Neoplasm positive sample. Bethesda IV FNA. (d) Hürthle positive, Neoplasm positive sample. Bethesda IV FNA. **Figure S2.** Comparing genome-wide LOH measured by CytoScan with LOH statistic from RNA-seq. **Figure S3.** Karyotype views of CytoScan data. Karyoview from Affymetrix’s Chromosome Analysis Suite (ChAS) software visualizing LOH (magenta), Gain (dark blue), Gain-Mosaicism (light blue), Loss (red), Loss-Mosaicism (light pink) events across 24 chromosomes. Chromosomes are shown in order from 1-22, X, and Y. Events spanning > 100 Kb are shown, and are indicated as a small mark (four-sided star for LOH, upward and downward triangle for gain and loss, respectively). (a) Hürthle cell adenoma sample exhibiting all five types of large aberrations: LOH on chromosomes (chr) 2, 3, 8, 9, and X; Gain and Gain-Mosaicism on chr 5, 7, 18, 19, 20, and Loss and Loss-Mosaicism on chr 1, 2, 3, 8, 9, 11, 13, 21, 22. (b) Hürthle cell carcinoma sample exhibiting large LOH or Gain events: LOH and Gain events alternatively occur on almost all chromosomes, except chr 14 where both events are present. (c) Normal sample. No large aberrations observed; only small LOH events are scattered around across the genome with limited Gain (chr 4 and chr 14) and Gain-Mosaicism (chr 4). (PDF 1097 kb)

## Abbreviations

AUC: Area under the curve
BFN: Benign follicular nodule
CLT: Chronic lymphocytic thyroiditis
CNV: Copy number variation
FA: Follicular adenoma
FNAB: Fine needle aspirate biopsy
GEC: Gene expression classifier
GSC: Genomic sequencing classifier
HCA: Hürthle cell adenoma
HCC: Hürthle cell carcinoma
HI: Hürthle cell index
LOH: Loss of heterozygosity
ML: Machine learning
MTC: Medullary thyroid carcinoma
NI: Hürthle cell Neoplasm Index
PTA: Parathyroid Adenoma
SNP: Single Nucleotide Polymorphism
SVM: Support vector machine
VAF: Variant Allele Frequency
