# Supplementary material for: Identification of Hürthle cell cancers: solving a clinical challenge with genomic sequencing and a trio of machine learning algorithms
Source: BMC Syst Biol. 2019 Apr 5;13(Suppl 2):27. doi: 10.1186/s12918-019-0693-z (PMC6450053; doi:10.1186/s12918-019-0693-z)
Supplement: Supplementary file 1 — Figure S1. Representative cytopathology images of Hürthle and Neoplasm samples. (a) Hürthle negative, Neoplasm negative sample. Bethesda II FNA. (b) Hürthle positive, Neoplasm negative sample. Bethesda II FNA. (c) Hürthle negative, Neoplasm positive sample. Bethesda IV FNA. (d) Hürthle positive, Neoplasm positive sample. Bethesda IV FNA. Figure S2. Comparing genome-wide LOH measured by CytoScan with LOH statistic from RNA-seq. Figure S3. Karyotype views of CytoScan data. Karyoview from Affymetrix’s Chromosome Analysis Suite (ChAS) software visualizing LOH (magenta), Gain (dark blue), Gain-Mosaicism (light blue), Loss (red), Loss-Mosaicism (light pink) events across 24 chromosomes. Chromosomes are shown in order from 1-22, X, and Y. Events spanning > 100 Kb are shown, and are indicated as a small mark (four-sided star for LOH, upward and downward triangle for gain and loss, respectively). (a) Hürthle cell adenoma sample exhibiting all five types of large aberrations: LOH on chromosomes (chr) 2, 3, 8, 9, and X; Gain and Gain-Mosaicism on chr 5, 7, 18, 19, 20, and Loss and Loss-Mosaicism on chr 1, 2, 3, 8, 9, 11, 13, 21, 22. (b) Hürthle cell carcinoma sample exhibiting large LOH or Gain events: LOH and Gain events alternatively occur on almost all chromosomes, except chr 14 where both events are present. (c) Normal sample. No large aberrations observed; only small LOH events are scattered around across the genome with limited Gain (chr 4 and chr 14) and Gain-Mosaicism (chr 4). (PDF 1097 kb) [file 12918_2019_693_MOESM1_ESM.pdf]

Supplementary document

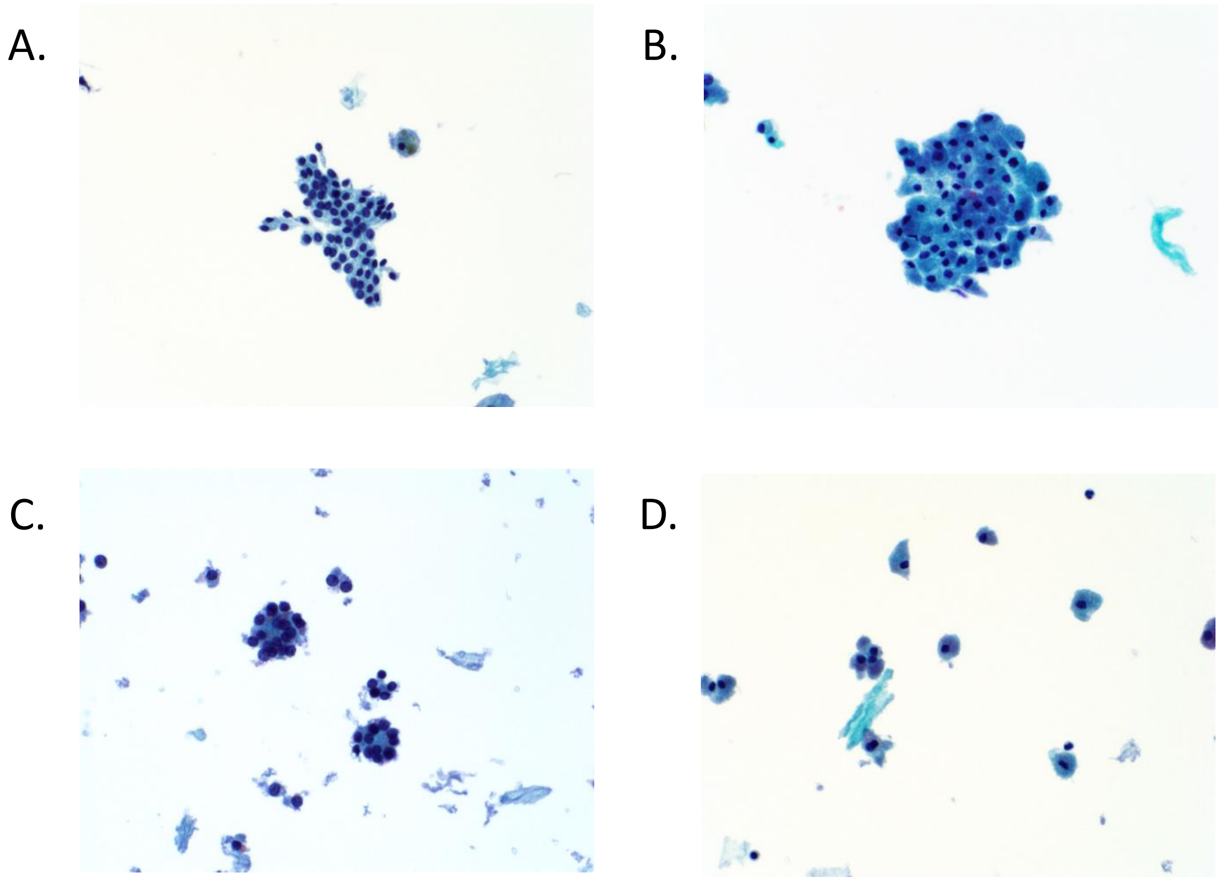

Figure S1. Representative cytopathology images of Hürthle and Neoplasm samples. (**a**) Hürthle negative, Neoplasm negative sample. Bethesda II FNA. (**b**) Hürthle positive, Neoplasm negative sample. Bethesda II FNA. (**c**) Hürthle negative, Neoplasm positive sample. Bethesda IV FNA. (**d**) Hürthle positive, Neoplasm positive sample. Bethesda IV FNA.

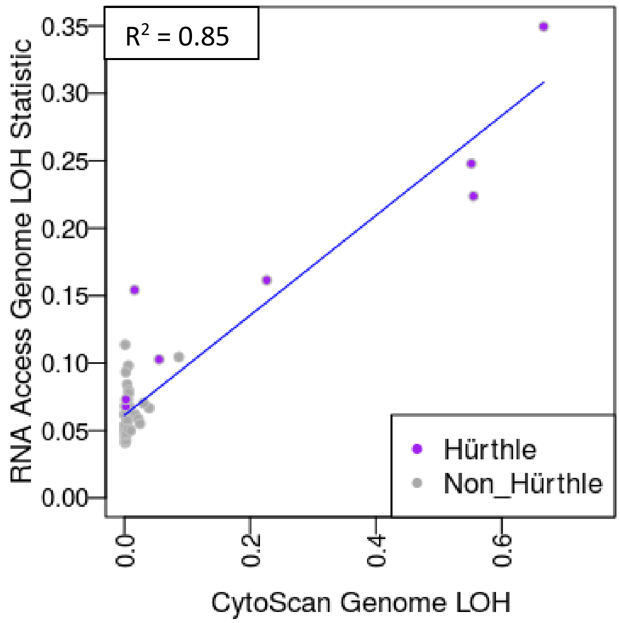

Figure S2. Comparing genome-wide LOH measured by CytoScan with LOH statistic from RNA-seq.

A.

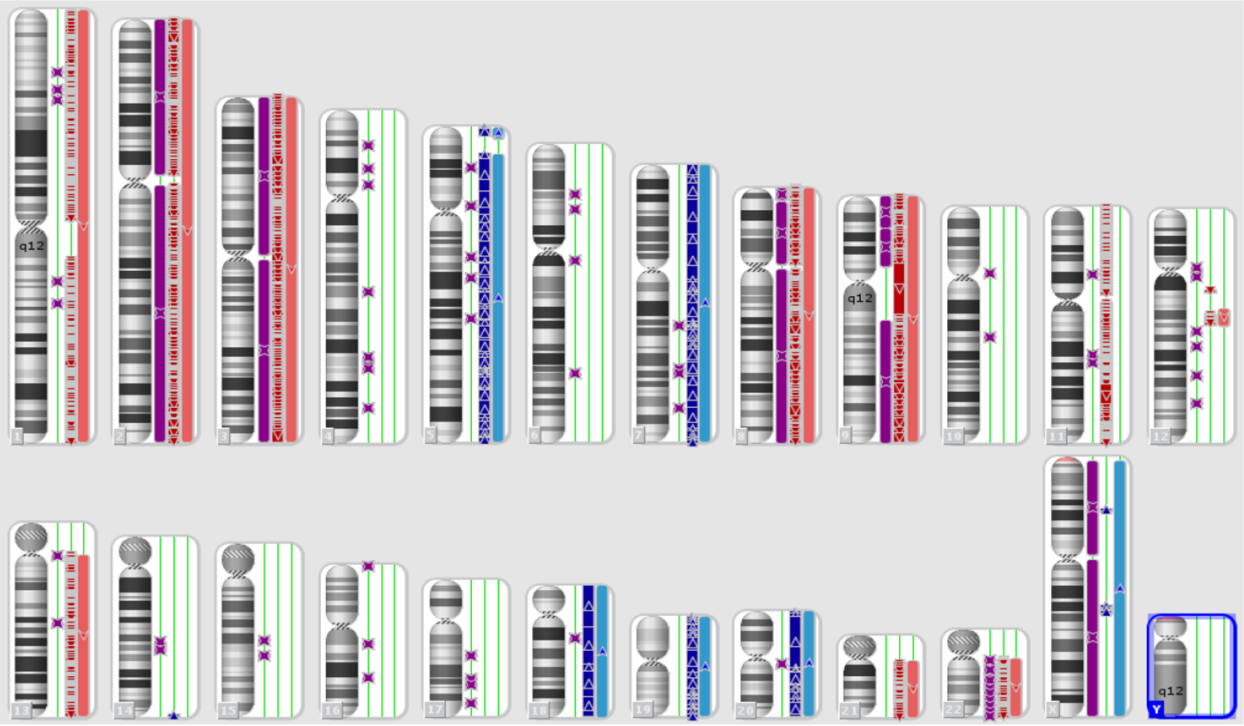

B.

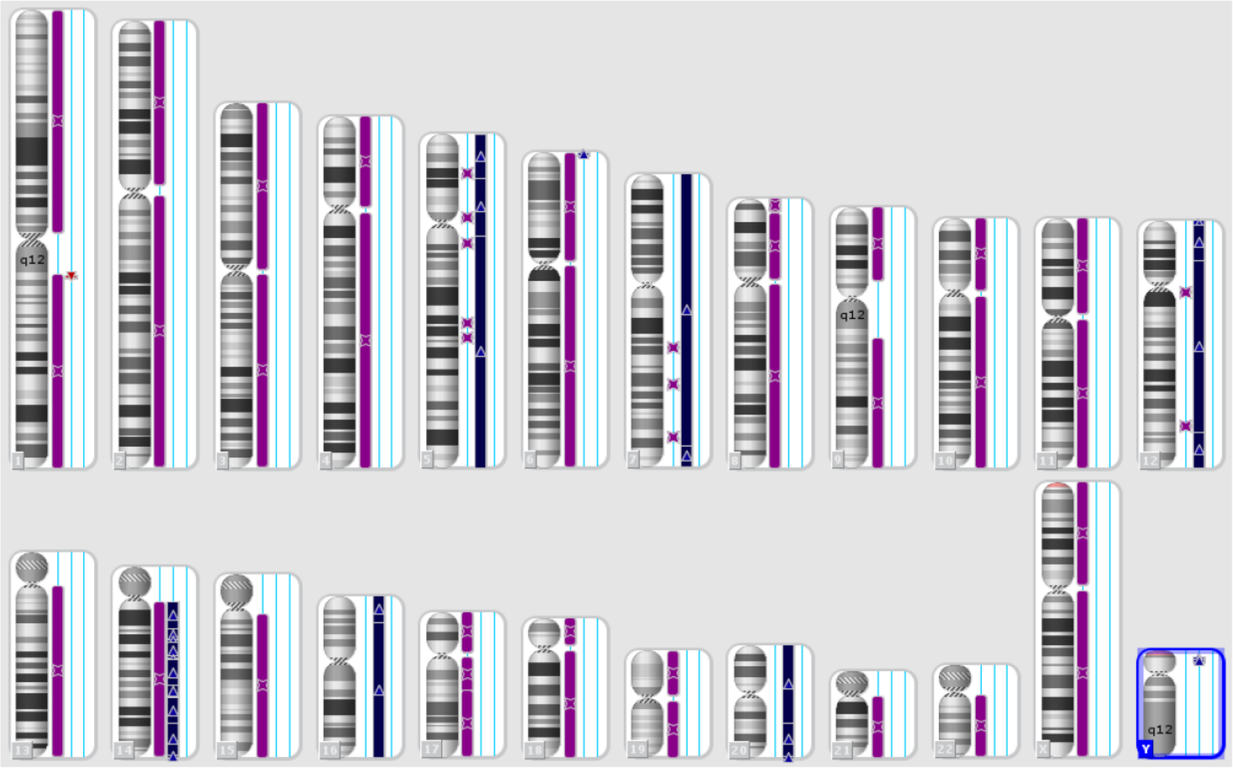

C.

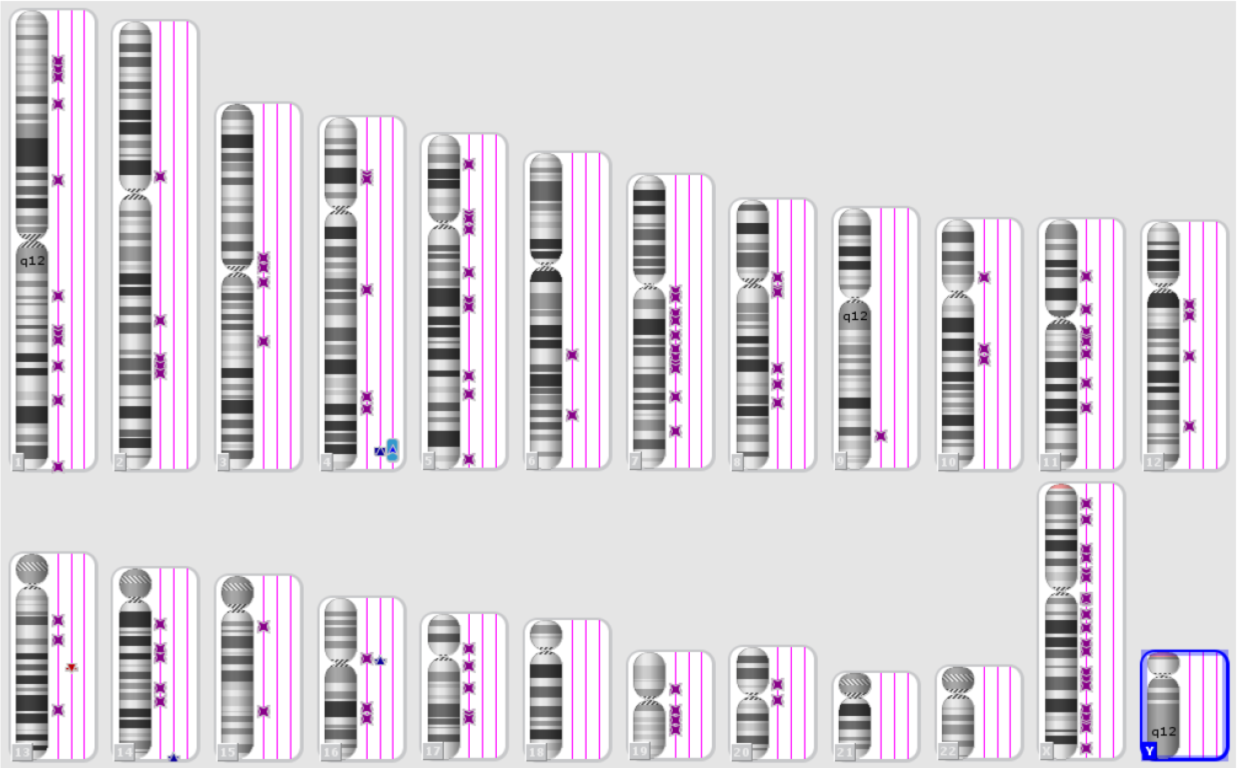

Figure S3. Karyotype views of CytoScan data. Karyoview from Affymetrix's Chromosome Analysis Suite (ChAS) software visualizing LOH (magenta), Gain (dark blue), Gain-Mosaicism (light blue), Loss (red), Loss-Mosaicism (light pink) events across 24 chromosomes. Chromosomes are shown in order from 1-22, X, and Y. Events spanning > 100 Kb are shown, and are indicated as a small mark (four-sided star for LOH, upward and downward triangle for gain and loss, respectively). (**a**) Hürthle cell adenoma sample exhibiting all five types of large aberrations: LOH on chromosomes (chr) 2, 3, 8, 9, and X; Gain and Gain-Mosaicism on chr 5, 7, 18, 19, 20, and Loss and Loss-Mosaicism on chr 1, 2, 3, 8, 9, 11, 13, 21, 22. (**b**) Hürthle cell carcinoma sample exhibiting large LOH or Gain events: LOH and Gain events alternatively occur on almost all chromosomes, except chr 14 where both events are present. (**c**) Normal sample. No large aberrations observed; only small LOH events are scattered around across the genome with limited Gain (chr 4 and chr 14) and Gain-Mosaicism (chr 4).
